# The High Expression of Minichromosome Maintenance Complex Component 5 Is an Adverse Prognostic Factor in Lung Adenocarcinoma

**DOI:** 10.1155/2022/4338793

**Published:** 2022-03-20

**Authors:** Man Sun, Tao Wang, Yonglin Zhu, Yanmei Zhang, Lichao Zhu, Xiaoxiao Li

**Affiliations:** Department of Geriatrics, The Second Affiliated Hospital of Zhengzhou University, No. 2 Jingba Road, Zhengzhou 450000, China

## Abstract

**Background:**

Minichromosome maintenance (MCM) genes are crucial for genomic DNA replication and are important biomarkers in tumor biology. In this study, we aimed to identify the diagnostic, therapeutic, and prognostic value of the MCM2–10 genes in patients with lung cancer.

**Methods:**

We examined the expression levels, gene networks, and protein networks of lung cancer using data from the ONCOMINE, GeneMANIA, and STRING databases. We conducted a functional enrichment analysis of MCM2–10 using the clusterProfiler package using TCGA data. The correlation between the MCM2–10 expression and lung cancer prognosis was evaluated using Cox regression analysis. The influence of clinical variables on overall survival (OS) was evaluated using univariate and multivariate analyses. The TIMER database was used to evaluate the correlation between tumor infiltrating levels and lung cancer. Kaplan–Meier Plotter pan-cancer RNA sequencing was used to estimate the correlation between the MCM5 expression and OS in different immune cell subgroups in patients with lung adenocarcinoma (LUAD). Finally, the 1-, 3-, and 5-year predictions of LUAD were performed using nomogram and calibration analysis.

**Results:**

The expression of MCM2, 3, 4, 5, 6, 7, 8, and 10 in lung cancer was higher than that for normal samples. The MCM5 expression was associated with poor OS in patients with LUAD, and prognosis was related to TNM stage, smoking status, and pathological stage. The MCM5 expression is correlated with immune invasion in LUAD and may affect prognosis due to immune infiltration.

**Conclusion:**

MCM5 may serve as a molecular biomarker for LUAD prognosis.

## 1. Introduction

Lung cancer is the leading cause of cancer-related morbidity and mortality worldwide [1]. Numerous studies have evaluated therapeutic approaches for reducing mortality rates in patients with lung adenocarcinoma (LUAD) [2]; however, the 5-year survival rate of patients with lung cancer from 2009 to 2015 was only 19% [3]. Further studies are required to identify accurate and promising prognostic biomarkers and efficient therapeutic targets to enhance survival rates in patients with lung cancer and to guide customized treatments [4].

The minichromosome maintenance (MCM) gene family plays key roles in DNA replication and cell cycle progression [5]. DNA replication errors can lead to tumorigenesis [6]. MCM family proteins are involved in the occurrence and development of cancer [7]. Indeed, several studies have shown that MCM proteins are highly expressed in various cancers, including pancreatic ductal adenocarcinoma [8], hepatocellular carcinoma [9], and colorectal cancer [10] and can be used as molecular markers for diagnosis and prognosis. Teresita et al. suggested that the progression of precancerous lung disease to carcinoma in situ is enhanced in MCM2-overexpressing cells [11]. MCM3 is involved in the carcinogenesis of multiple cancers [12] and is associated with the development of LUAD [13]. Yi et al. identified MCM4 as a potential lung cancer driver gene and demonstrated that MCM4 upregulation is associated with poorer survival in patients with lung cancer [14]. MCM6 levels are higher in primary lung tumors with both FHIT and p53 inactivation [15]. MCM7 is involved in tumor formation, progression, malignant transformation, and prognosis [16] and can be used as a potential biomarker for the poor prognosis of non-small-cell lung cancer [17]. MCM9 is an outlier within the MCM family, containing a long C-terminal extension comprising 42% of the total length, but with no known functional components and high predicted disorder [18]. MCM10 acts as an oncogene that promotes the progression of hepatocellular carcinoma [19]. However, a correlation between the MCM2–10 gene expression and immune infiltration in lung cancer has rarely been reported.

Accordingly, in this study, bioinformatic methods were used to analyze online public databases to assess the expression of MCM2–10 genes in patients with lung cancer and the relationship between this expression and tumor prognosis. Our findings may contribute to the screening, diagnosis, treatment, and prognosis of patients with lung cancer.

## 2. Materials and Methods

### 2.1. ONCOMINE and the Cancer Genome Atlas (TCGA)

ONCOMINE (http://www.oncomine.org/) is a tumor microarray database with functions for differential gene expression analysis, correlation analysis between gene expression and clinical features, prognostic analysis, and multigene coexpression analysis [20, 21]. The differential expression of MCM2–10 in lung cancer was measured using Student's *t*-test (*p* < 0.01, fold change: 1.5, gene rank: 10%, data type: mRNA). We used paired sample *t*-test analysis TCGA (https://portal.gdc.cancer.gov/) LUADLUSC (lung cancer) [22] in the project level 3 HTSeq-RNAseq FPKM format data to assess target genes in lung cancer and normal tissues (ns, *p* ≥ 0.05; ^∗^*p* < 0.05; ^∗∗^*p* < 0.01; ^∗∗∗^*p* < 0.001).

### 2.2. Networks of MCM2–10 Interacting Genes and Proteins

GeneMANIA (http://www.genemania.org) is useful for predicting the function of MCM2–10. The STRING database (version 11.5; https://string-db.org/) was used to determine the protein-protein interactions of MCM2–10 [23].

### 2.3. Functional Enrichment and KEGG Pathway Analysis of MCM2–10

Gene Ontology (GO) functional annotation was performed using biological processes (BP), cellular components (CC), and molecular functions (MF). The Kyoto Encyclopedia of Genes and Genomes (KEGG) (https://www.kegg.jp/kegg/) pathway is useful for understanding molecular interactions, reactions, genetic information processing, environmental information processing, cellular processes, and human diseases. The following *R* packages were used: clusterProfiler package (version 3.14.3) for GO and KEGG enrichment analyses and ggplot2 package (version 3.3.3) for visualization [24].

### 2.4. cBioPortal for Cancer Genomics

The cBio Cancer Genomics Portal (cBioPortal; http://cbioportal.org) utilizes data from more than 5,000 tumor samples from 20 cancer studies to provide a web resource for exploring, visualizing, and analyzing multidimensional cancer genomics data [25]. We investigated Pan Lung Cancer (TCGA, Nat Genet 2016) [26] data to explore genetic alterations in MCM2–10.

### 2.5. The Prognostic Value of MCMs in Patients with Lung Cancer

The correlation between the MCM2–10 expression and lung cancer prognosis was evaluated by Cox regression analysis of TCGA data [27], the survminer package (version 0.4.9) for visualization, and the survival package (version 3.2-10) for statistical analysis of survival data. The influence of the clinical variables on overall survival (OS) was evaluated using univariate and multivariate analyses. Kaplan–Meier Plotter pan-cancer RNA-sequencing (RNA-seq) [28] was used to estimate the correlation between the MCM5 expression and OS in different immune cell subgroups of patients with LUAD. Clinical variables (smoking status, pathological stage, primary therapy outcome, and MCM5 expression) were analyzed using the rms package (version 6.2-0) and survival package (version 3.2-10) to predict the 1-, 3-, and 5-year OS of patients with LUAD.

### 2.6. Intergroup Comparison of the MCM5 Gene Expression and Tumor Clinical Variables

The Wilcoxon rank-sum test was used to compare the tumor and normal lung tissue groups. The Kruskal–Wallis test was used for intergroup comparison of TNM stage, pathological stage, sex, age, smoking status, primary therapy outcome, and overall survival (OS), progression free interval (PFI), and disease free survival (DSS) events (ns, *p* ≥ 0.05; ^∗^*p* < 0.05; ^∗∗^*p* < 0.01; ^∗∗∗^*p* < 0.001).

### 2.7. Tumor Immune Estimation Resource (TIMER)

TIMER (https://cistrome.shinyapps.io/timer/) was used to evaluate the correlation between tumor-infiltrating levels in lung cancer and alterations of different somatic copy numbers in MCM5 [29, 30]. The correlation between the MCM5 expression and six immune infiltrates (B cells, CD4+ T cells, CD8+ T cells, neutrophils, macrophages, and dendritic cells [DCs]) was estimated using the TIMER algorithm. The sGSVA package (version 1.34.0) [31] and Spearman correlation analysis were applied to correlate MCM5 and B cells, CD8+ T cells, cytotoxic cells, DC, eosinophils, immature DCs, macrophages, mast cells, neutrophils, natural killer (NK) CD56 bright cells, NK CD56 dim cells, NK cells, plasmacytoid DCs, T cells, T helper cells, T central memory, T effector memory, T follicular helper, T gamma delta (Tgd), Th1 cells, Th17 cells, Th2 cells, and regulatory T (Treg) cells [32].

## 3. Results

### 3.1. The Overexpression of Different MCM2–10 Genes in Lung Cancer

**Figure 1:**
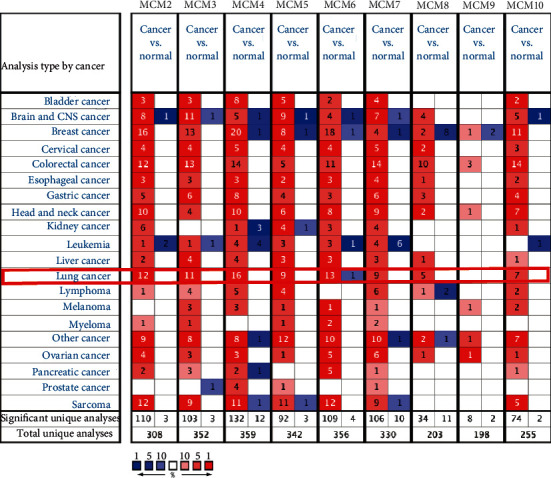
The mRNA expression of MCM2-10 in 20 kinds of cancer (ONCOMINE). MCM2-8 and MCM10 were highly expressed in lung cancer. The difference was compared by Students' *t*-test, *p* value: 0.01, fold change: 1.5, gene rank: 10%, data type: mRNA. Red represents high expression, and blue represents low expression.

**Figure 2:**
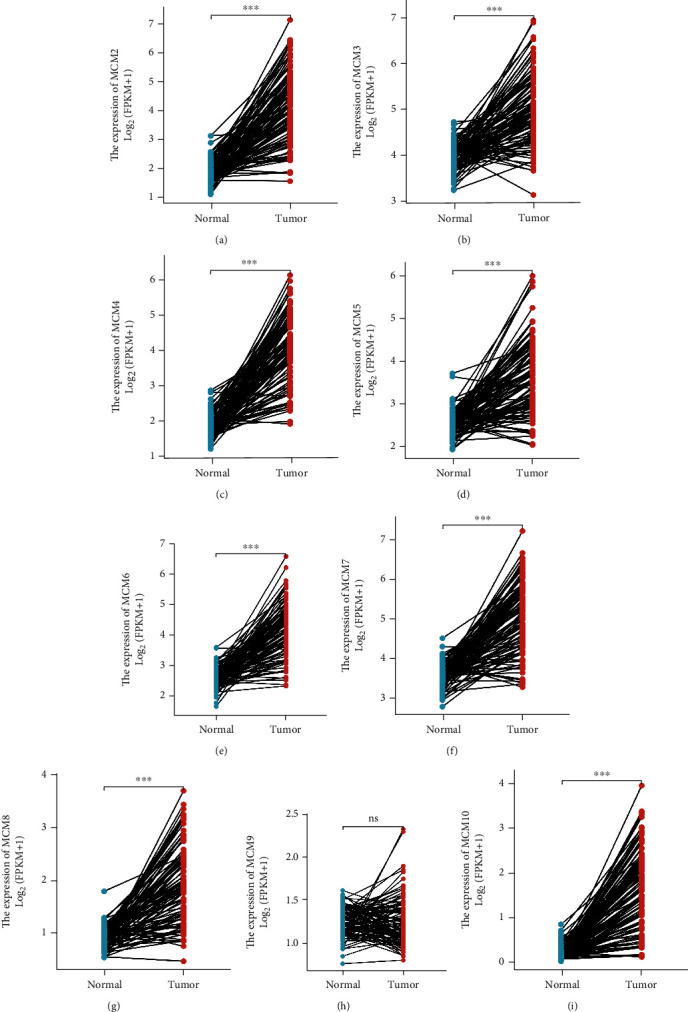
The expression of MCM2-10 in paired lung cancer and normal lung tissues. The high expression of MCM2-8 and MCM10 was observed in lung cancer samples analyzed by paired sample *t*-test analysis, ns, *p* ≥ 0.05; ^∗^*p* < 0.05; ^∗∗^*p* < 0.01; ^∗∗∗^*p* < 0.001.

The transcriptional expression of MCM2–10 genes in lung cancer and normal samples was investigated using the ONCOMINE database (http://www.oncomine.org/); Figure 1, Table 1). In the data investigated, MCM genes showed overall overexpression in lung cancer. The fold change varied, with the highest fold change of 3.251 for MCM2 [33], 1.617 for MCM3 [34], 2.649 for MCM4 [35], 1.810 for MCM5 [36], 1.797 for MCM6 [34], 1.628 for MCM7 [37], 1.431 for MCM8 [38], 1,733 for MCM10 [34] in LUAD, and 6.171 for MCM2 [39], 2.387 for MCM3 [36], 3.108 for MCM4 [36], 4.682 for MCM5 [40], 2.650 for MCM6 [33], 2.691 for MCM7 [33], 3.587 for MCM8 [33], and 4.099 for MCM10 [33] in lung squamous cell carcinoma (LUSC). A *t*-test of paired samples showed that the expression of MCM2, 3, 4, 5, 6, 7, 8, and 10 in lung cancer was higher than the average level of normal, and the difference was statistically significant (*p* < 0.001; Figure 2).

### 3.2. Functional Enrichment of MCM2–10 in Patients with Lung Cancer

**Figure 3:**
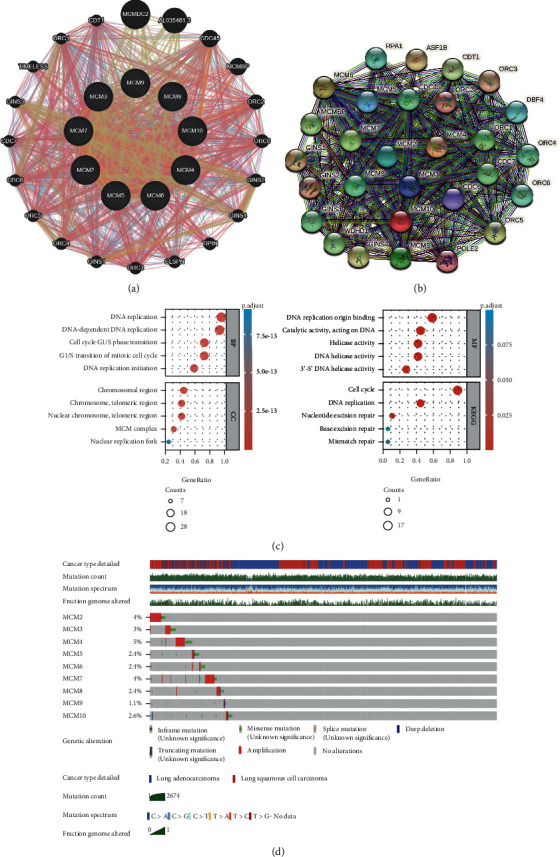
The gene-gene and protein-protein interaction network, functional enrichment, and genomic alterations of MCM2-10. (a) The gene-gene network associated with the MCM2-10 (GeneMANIA). (b) The protein-protein network of MCM2-10 (STRING). (c) The GO and KEGG enrichment of MCM2-10. (d) Alteration frequency of MCM2-10 in lung cancer patients (cBioPortal). GO: Gene Ontology; KEGG: Kyoto Encyclopedia of Genes and Genomes (KEGG).

Gene-gene interaction (Figure 3(a)) and protein-protein networks (Figure 3(b)) of MCM2–10 were constructed. The functional enrichment of 30 molecules obtained from the protein-protein network was predicted using the clusterProfiler package. GO terms were analyzed according to BP, MF, and CC (Figure 3(c) and Supplemental Table 1). The BP associated with MCM2–10 included DNA-dependent DNA replication, DNA replication, and DNA replication initiation. The MF were associated with DNA replication origin binding, DNA helicase activity, 3′-5′-DNA helicase activity, catalytic activity, acting on DNA, and helicase activity. The CC were associated with MCM complex, nuclear chromosome, telomeric region, chromosome, telomeric region, chromosomal region, and nuclear replication fork. In the KEGG analysis, five pathways were associated with MCM2–10, and the cell cycle pathway accounted for the highest proportion. The cBioPortal online tool was then used to evaluate the frequency of MCM2–10 alteration in lung cancer. In total, 1144 samples from TCGA were analyzed, and the percentage of genetic alterations in MCM2–10 for lung cancer varied from 1.1% to 5% (Figure 3(d)).

### 3.3. Clinical Value of MCM5 in Lung Cancer

**Figure 4:**
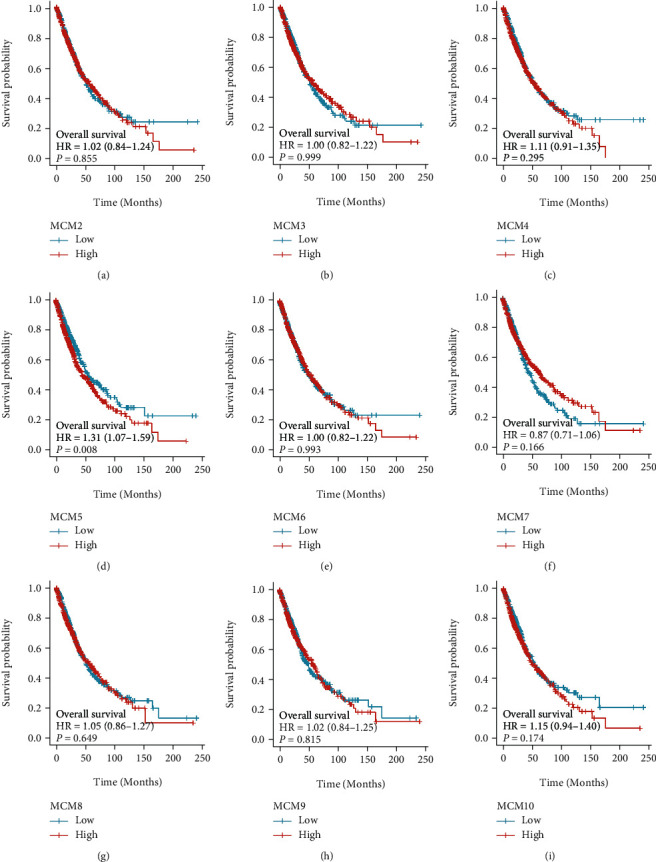
Correlation analysis of the abnormal MCM2-10 expression and overall survival in patients with lung cancer. (d) The mRNA expression of MCM5 was significantly associated with worse OS in patients with lung cancer, HR = 1.31 (1.07-1.59), *p* = 0.008. Red represents high expression, and blue represents low expression.

**Figure 5:**
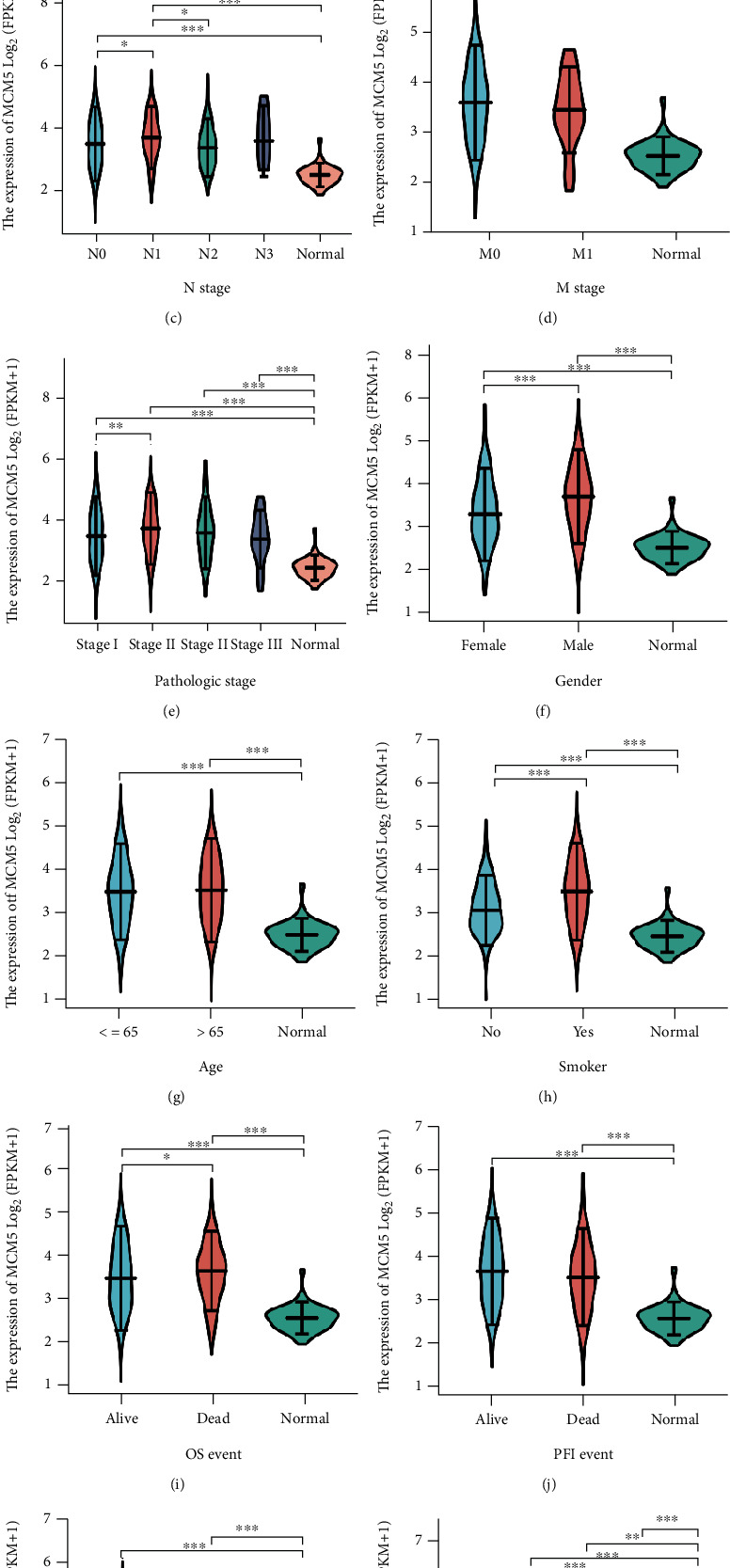
The expression of MCM5 in different clinical features of lung cancer. MCM5 was highly expressed in lung cancer and closely related to TNM stage, pathologic stage, sex, age, smoker, OS event, PFI event, DSS event, and primary therapy outcome, ns, *p* ≥ 0.05; ^∗^*p* < 0.05; ^∗∗^*p* < 0.01; ^∗∗∗^*p* < 0.001. OS: overall survival; PFI: progression-free interval; DSS: disease-specific survival; PD: progressive disease; SD: stable disease; PR: partial response; CR: complete response.

**Figure 6:**
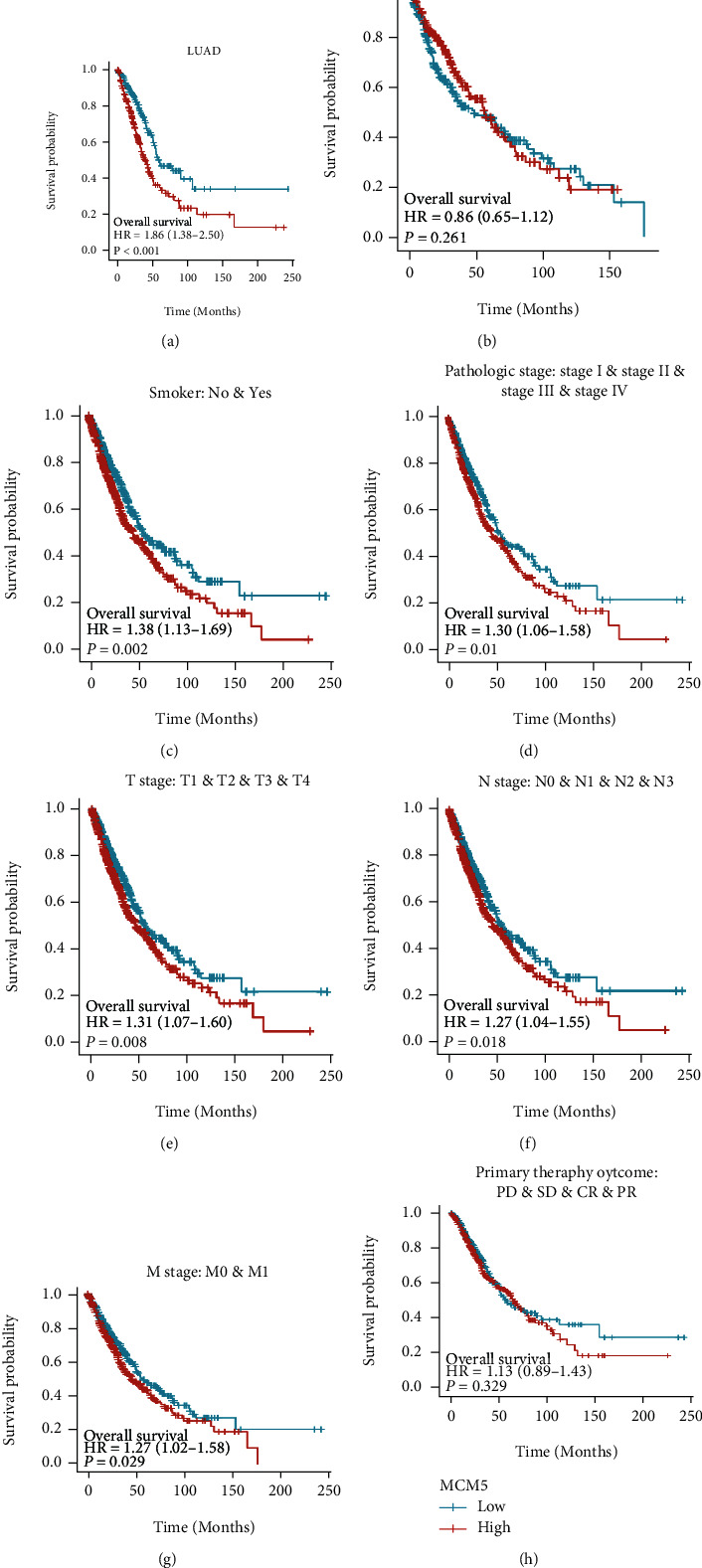
Cox regression survival analysis of overall survival (OS) probabilities concerning the MCM5 expression in lung cancer patients of different subgroups. The prognosis of lung cancer patients with high expression of MCM5 was related to lung adenocarcinoma (LUAD) (*p* < 0.001), smoker (*p* = 0.002), pathologic stage (*p* = 0.01), T stage (*p* = 0.008), N stage (*p* = 0.018), and M stage (*p* = 0.029).

We explored the prognostic value of MCM genes in the OS of patients with lung cancer. The mRNA expression of MCM5 (*p* = 0.008) was closely linked to worse OS in patients with lung cancer (Figure 4(d), Table 2). MCM5 was highly expressed in patients with lung cancer and was closely related to TNM stage, pathological stage, sex, age, smoking status, primary therapy outcomes, and OS, PFI, and DSS events (Figure 5 and Supplemental Table 2). Furthermore, we explored the correlation between MCM5 expression and clinicopathological parameters on OS in patients with lung cancer, and poor OS was associated with LUAD, TNM stage, smoking status, and pathological stage (Figures 6(a) and 6(c)–6(g)).

### 3.4. Correlation between the MCM5 Expression and Immune Infiltration Level

**Figure 7:**
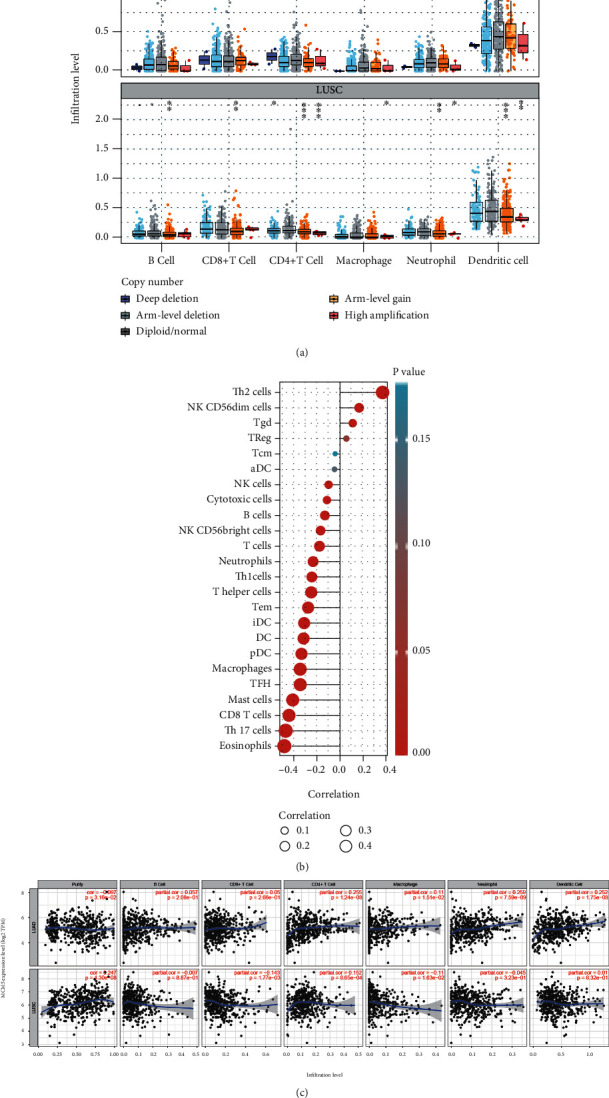
Correlations between the MCM5 expression and immune cells. (a) Correlation of tumor infiltrating levels in lung cancer and different somatic copy numbers' alterations in the MCM5 expression. (b) Correlations between the MCM5 expression and 24 immune cells. (c) MCM5 was positively correlated with infiltration levels of B cells, CD4+ T cells, CD8+ T cells, neutrophils, macrophages, and dendritic cells in LUAD and positively correlated with CD4+ T cells and dendritic cells in LUSC.

The TIMER online tool was used to investigate the correlation between the MCM5 expression and immune cell infiltration in lung cancer. The somatic copy number alteration module showed that the arm-level gain of MCM5 was significantly associated with immune cell infiltration levels in LUAD and LUSC (Figure 7(a)). MCM5 was positively correlated with the infiltration levels of Th2, NK CD56dim, Tgd, and Treg cells in lung cancer (Figure 7(b)). The MCM5 expression was positively correlated with the infiltration levels of B cells, CD4+ T cells, CD8+ T cells, neutrophils, macrophages, and DCs in LUAD (Figure 7(c)). MCM5 was positively correlated with infiltration levels of CD4+ T cells and DCs in LUSC (Figure 7(c)).

### 3.5. Prognostic Analysis of the MCM5 Expression Based on Immune Cells in LUAD Patients and Prognostic Predictive Model

**Figure 8:**
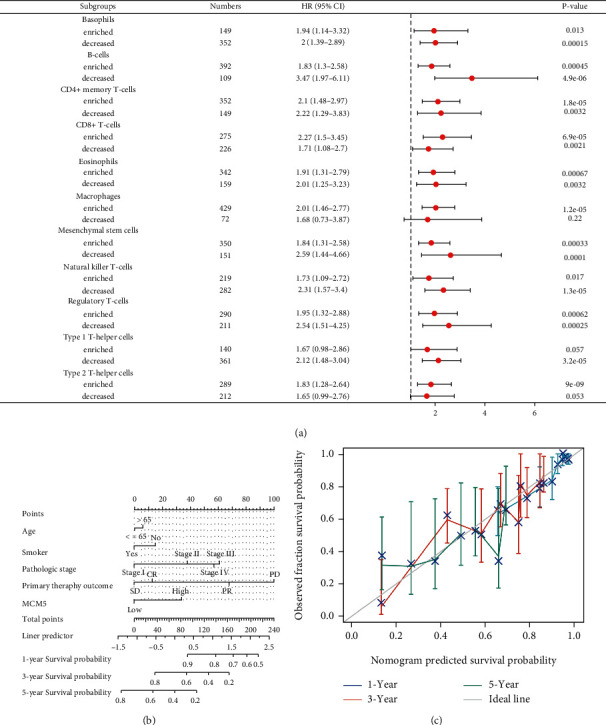
Relationship between MCM5 and immune infiltration with overall survival (OS). (a) A forest plot shows the prognostic value of MCM5 expression according to different immune cell subgroups in LUAD patients. (b) Nomogram for predicting the probability of 1-, 3-, and 5-year OS for patients with LUAD. (c) Calibration plot of the nomogram for predicting the OS likelihood.

The high MCM5 expression was closely related to LUAD prognosis and immune cell infiltration. We further explored whether the high MCM5 expression affected the prognosis because of immune infiltration. The Kaplan–Meier Plotter pan-cancer RNA-seq LUAD (*n* = 513) data were analyzed for the prognosis of enriched and decreased immune cells. Poor OS was seen in LUAD patients with the high MCM5 expression and enriched infiltration of basophils, B cells, CD4+ memory T cells, CD8+ T cells, eosinophils, macrophages, mesenchymal stem cells, natural killer T cells, Treg cells, and type 2 T-helper cells, and in LUAD patients with the high MCM5 expression and decreased infiltration of basophils, B cells, CD4+ memory T cells, eosinophils, mesenchymal stem cells, natural killer T cells, Treg cells, and type 1 T-helper cells. Enriched type 1 T-helper cells, decreased macrophages, and type 2 T-helper cells showed no significant correlation between the MCM5 expression and OS in patients with LUAD (Figure 8(a)). These findings reveal that MCM5 may affect the prognosis of patients with LUAD, in part due to immune infiltration. Finally, we used nomogram and calibration analysis to predict the 1-, 3-, and 5-year OS of patients with LUAD using clinically related factors such as age, smoking status, pathological stage, and primary therapy outcome (Figures 8(b) and 8(c)).

## 4. Discussion

Recent studies have suggested that dysregulation of MCMs leads to tumor initiation, progression, and chemoresistance via modulation of the cell cycle and DNA replication stress [41]. The MCM protein plays a key role in the proliferation and prognosis of lung cancer [16]. Bioinformatic analysis was used to detect mRNA expression, prognostic value, genetic mutations, functional enrichment, protein-protein network, and immune infiltration of MCMs in patients with lung cancer.

MCM2 plays a role in the proliferation, circulation, and migration of lung cancer cells [42]. MCM3 regulates cell proliferation by binding to cyclin D1 [43]. Mutations in MCM4 disrupt the functions of MCM2–7, resulting in genomic instability and cancer progression [44]. MCM5 is an important DNA replication initiation factor and is strongly downregulated following the overexpression of the long noncoding RNA CARMN [45]. MCM6, MCM7, and MCM8 collaborate with other MCM family members to promote cancer cell proliferation through cell cycle and DNA replication [46, 47]. The MCM9 protein is involved in the unwinding activity [48]. MCM10 mediates DNA replication by collaborating with other cell-dividing cyclins [49]. The BP and associated pathways of MCM2-10 were elucidated by GO and KEGG enrichment analyses, which are useful for investigating the pathological mechanisms of lung cancer. The BP associated with MCM2–10 includes DNA-dependent DNA replication, DNA replication, DNA replication initiation, G1/S transition of mitotic cell cycle, and cell cycle G1/S phase transition. The cell cycle and DNA replication pathways are associated with MCM2–10. MCM2-8 and MCM10 were highly expressed in paired lung cancer samples and may be involved in the development of lung cancer through the cell cycle and DNA replication.

Correlation analysis between the MCM2-10 expression and OS revealed that only MCM5 was closely related to poor OS in patients with lung cancer. The MCM5 gene affects the prognosis of LUAD by regulating BP and pathways, such as cell cycle and DNA replication [50]. In this study, MCM5 was highly expressed in tumors, which is related to TNM stage, pathological stage, sex, age, smoking status, prognostic events, and primary therapy outcomes. MCM5 was positively correlated with poor OS in patients with LUAD and was influenced by TNM stage, smoking status, age, and pathological stage. These results suggest that MCM5 is involved in the development of lung cancer, may be used as a molecular target for diagnosis and treatment, and is an independent prognostic marker of lung cancer. Furthermore, we revealed that the arm-level gain of MCM5 was significantly associated with immune cell infiltration levels in lung cancer. MCM5 positively correlated with B cells, CD4+ T cells, CD8+ T cells, neutrophils, macrophages, and DCs in LUAD. Enriched type 1 T-helper cells, decreased macrophages, and type 2 T-helper cells showed no significant correlation between the MCM5 expression and OS in patients with LUAD, whereas decreased type 1 T-helper cells, enriched macrophages, and type 2 T helper cells were related to the OS of patients with LUAD. MCM5 may partially influence the OS of patients with LUAD by immune cell infiltration. However, the exact role of MCM5 in the tumor immune microenvironment requires further investigation. Furthermore, we revealed that the arm-level gain of MCM5 was significantly associated with immune cell infiltration levels in lung cancer. MCM5 positively correlated with the infiltration levels of B cells, CD4+ T cells, CD8+ T cells, neutrophils, macrophages, DCs, and partially influenced the OS of patients with LUAD by immune cell infiltration. Thus, MCM5 may serve as a molecular biomarker for immunotherapy. However, the exact role of MCM5 in the tumor immune microenvironment requires further exploration.

Our study had certain limitations. The data were collected online from open databases. In future studies, large clinical datasets are required to verify our findings, and the role of MCM2–10 in the pathogenesis of lung cancer should be further explored.

## 5. Conclusions

Our findings demonstrated that MCM2-8 and 10 were highly expressed in lung cancer, and only MCM5 affected the prognosis of patients with lung cancer. The influence of MCM5 on the prognosis of lung cancer patients was related to LUAD, smoking, pathologic stage, and TNM stages. We further confirmed that the abnormal expression of MCM5 in LUAD was related to immune cell infiltration, and immune cell infiltration may contribute to the prognosis of LUAD partly. The above findings suggested that MCM5 can be used as a molecular marker for the prognosis of LUAD.

## Figures and Tables

**Table 1:** The mRNA expression of MCM2-10 was significantly expressed in lung cancer (ONCOMINE).

	Type of lung cancer vs. normal	Fold change	*p* value	*t*-test	Ref
MCM2	Lung adenocarcinoma Lung adenocarcinoma Squamous cell lung carcinoma Squamous cell lung carcinoma	1.993 3.251 6.171 2.204	7.61*E*-17 3.46*E*-13 1.99*E*-05 1.13*E*-11	10.52 9.295 12.132 8.803	Landi et al. [37] Hou et al. [33] Wachi et al. [39] Talbot et al. [51]

MCM3	Lung adenocarcinoma Lung adenocarcinoma Squamous cell lung carcinoma Squamous cell lung carcinoma	1.617 1.047 1.653 2.387	4.13*E*-16 3.39*E*-07 1.33*E*-10 8.85*E*-05	9.920 5.093 7.742 4.773	Selamat et al. [34] TCGA Talbot et al. [51] Garber et al. [36]

MCM4	Lung adenocarcinoma Lung adenocarcinoma Lung adenocarcinoma Lung adenocarcinoma Squamous cell lung carcinoma Squamous cell lung carcinoma	2.403 2.649 1.668 1.100 3.108 1.101	8.50*E*-19 7.09*E*-10 6.17*E*-12 3.68*E*-16 4.84*E*-07 1.12*E*-22	11.190 8.123 8.670 8.605 8.298 10.441	Landi et al. [37] Su et al. [35] Okayama et al. [38] TCGA Garber et al. [36] TCGA

MCM5	Lung adenocarcinoma Lung adenocarcinoma Squamous cell lung carcinoma Squamous cell lung carcinoma	1.810 1.367 4.682 1.072	4.58*E*-06 4.16*E*-06 7.91*E*-07 7.43*E*-15	5.433 5.442 5.941 8.033	Garber et al. [36] Beer et al. [52] Bhattacharjee et al. [40] TCGA

MCM6	Lung adenocarcinoma Squamous cell lung carcinoma Squamous cell lung carcinoma	1.797 2.650 1.023	2.63*E*-12 6.05*E*-17 5.34*E*-05	8.130 12.633 3.920	Selamat et al. [34] Hou et al. [33] TCGA

MCM7	Lung adenocarcinoma Squamous cell lung carcinoma	1.628 2.691	2.08*E*-10 5.94*E*-15	7.057 13.573	Landi et al. [37] Hou et al. [33]

MCM8	Lung adenocarcinoma Lung adenocarcinoma Squamous cell lung carcinoma	1.322 1.431 3.587	6.39*E*-12 3.51*E*-08 6.27*E*-12	8.202 6.860 10.719	Selamat et al. [34] Okayama et al. [38] Hou et al. [33]

MCM9	NA				
MCM10	Lung adenocarcinoma Squamous cell lung carcinoma	1.733 4.099	5.36*E*-14 4.06*E*-16	9.601 14.598	Selamat et al. [34] Hou et al. [33]


**Table 2:** Univariate and multivariate analysis of the correlation of MCM5 expression with OS in lung cancer patients.

Characteristics	Total (*N*)	Univariate analysis		Multivariate analysis	
		Hazard ratio (95% CI)	*p* value	Hazard ratio (95% CI)	*p* value
T stage	996				
T1	282	Reference			
T2	557	1.375 (1.073-1.764)	0.012	1.216 (0.900-1.642)	0.203
T3 and T4	157	2.360 (1.742-3.197)	<0.001	1.831 (1.149-2.919)	0.011
N stage	981				
N0	641	Reference			
N1	222	1.527 (1.212-1.922)	<0.001	1.144 (0.761-1.722)	0.518
N2 and N3	118	2.018 (1.522-2.675)	<0.001	2.025 (1.119-3.666)	0.020
M stage	774				
M0	742	Reference			
M1	32	2.250 (1.427-3.548)	<0.001	2.063 (1.128-3.771)	0.019
Age	983				
< =65	427	Reference			
>65	556	1.259 (1.027-1.544)	0.027	1.368 (1.083-1.730)	0.009
Pathologic stage	987				
Stage I	512	Reference			
Stage II	278	1.605 (1.264-2.038)	<0.001	1.187 (0.769-1.833)	0.439
Stage III	164	2.239 (1.730-2.898)	<0.001	0.989 (0.513-1.908)	0.975
Stage IV	33	3.076 (1.947-4.859)	<0.001		
MCM5	999				
Low	501	Reference			
High	498	1.274 (1.045-1.554)	0.017	1.216 (0.968-1.526)	0.093

## Data Availability

The data in this paper were mined from online public databases.

## Supplementary Materials

Supplementary 1 C:\Users\86137\Desktop\supplemental Table 1.html.

Supplementary 2 C:\Users\86137\Desktop\supplemental Table 2.html.
