# Supplementary material for: The High Expression of Minichromosome Maintenance Complex Component 5 Is an Adverse Prognostic Factor in Lung Adenocarcinoma
Source: Biomed Res Int. 2022 Mar 20;2022:4338793. doi: 10.1155/2022/4338793 (PMC8961428; doi:10.1155/2022/4338793)
Supplement: Supplementary 1 — C:\Users\86137\Desktop\supplemental Table 1.html. [file 4338793.f1.docx]

| **Supplemental Table 1** The GO and KEGG enrichment of MCM2-10. | | | | | | | |
| --- | --- | --- | --- | --- | --- | --- | --- |
| ONTOLOGY | ID | Description | GeneRatio | BgRatio | pvalue | p.adjust | qvalue |
| BP | GO:0006261 | DNA-dependent DNA replication | 27/29 | 153/18670 | 1.65e-55 | 3.76e-53 | 1.65e-53 |
| BP | GO:0006260 | DNA replication | 28/29 | 274/18670 | 3.24e-51 | 3.69e-49 | 1.62e-49 |
| BP | GO:0006270 | DNA replication initiation | 17/29 | 37/18670 | 7.18e-41 | 5.46e-39 | 2.39e-39 |
| BP | GO:0000082 | G1/S transition of mitotic cell cycle | 21/29 | 279/18670 | 8.31e-33 | 4.74e-31 | 2.08e-31 |
| BP | GO:0044843 | cell cycle G1/S phase transition | 21/29 | 298/18670 | 3.46e-32 | 1.58e-30 | 6.92e-31 |
| CC | GO:0042555 | MCM complex | 9/29 | 12/19717 | 1.77e-24 | 5.50e-23 | 2.61e-23 |
| CC | GO:0000784 | nuclear chromosome, telomeric region | 12/29 | 125/19717 | 1.16e-19 | 1.80e-18 | 8.57e-19 |
| CC | GO:0000781 | chromosome, telomeric region | 12/29 | 161/19717 | 2.67e-18 | 2.76e-17 | 1.31e-17 |
| CC | GO:0098687 | chromosomal region | 13/29 | 349/19717 | 7.05e-16 | 5.46e-15 | 2.60e-15 |
| CC | GO:0043596 | nuclear replication fork | 7/29 | 41/19717 | 1.48e-13 | 9.17e-13 | 4.36e-13 |
| MF | GO:0003688 | DNA replication origin binding | 17/29 | 24/17697 | 3.91e-45 | 1.21e-43 | 3.71e-44 |
| MF | GO:0003678 | DNA helicase activity | 12/29 | 81/17697 | 1.76e-21 | 2.73e-20 | 8.33e-21 |
| MF | GO:0043138 | 3'-5' DNA helicase activity | 8/29 | 20/17697 | 2.24e-18 | 2.32e-17 | 7.08e-18 |
| MF | GO:0140097 | catalytic activity, acting on DNA | 13/29 | 213/17697 | 4.41e-18 | 3.42e-17 | 1.04e-17 |
| MF | GO:0004386 | helicase activity | 12/29 | 163/17697 | 1.12e-17 | 6.95e-17 | 2.12e-17 |
| KEGG | hsa04110 | Cell cycle | 16/18 | 124/8076 | 5.26e-28 | 3.68e-27 | 2.21e-27 |
| KEGG | hsa03030 | DNA replication | 8/18 | 36/8076 | 2.87e-15 | 1.00e-14 | 6.04e-15 |
| KEGG | hsa03420 | Nucleotide excision repair | 2/18 | 47/8076 | 0.005 | 0.011 | 0.007 |
| KEGG | hsa03430 | Mismatch repair | 1/18 | 23/8076 | 0.050 | 0.088 | 0.053 |
| KEGG | hsa03410 | Base excision repair | 1/18 | 33/8076 | 0.071 | 0.100 | 0.060 |
