# Supplementary material for: The High Expression of Minichromosome Maintenance Complex Component 5 Is an Adverse Prognostic Factor in Lung Adenocarcinoma
Source: Biomed Res Int. 2022 Mar 20;2022:4338793. doi: 10.1155/2022/4338793 (PMC8961428; doi:10.1155/2022/4338793)
Supplement: Supplementary 2 — C:\Users\86137\Desktop\supplemental Table 2.html. [file 4338793.f2.docx]

| **Supplemental Table 2**  Clinicopathologic characteristics of MCM5. | | | | |
| --- | --- | --- | --- | --- |
| Characteristic | levels | Low expression of MCM5 | High expression of MCM5 | p |
| n |  | 507 | 507 |  |
| T stage, n (%) | T1 | 164 (16.2%) | 118 (11.7%) | 0.006 |
|  | T2 | 265 (26.2%) | 304 (30.1%) |  |
|  | T3 | 60 (5.9%) | 58 (5.7%) |  |
|  | T4 | 16 (1.6%) | 26 (2.6%) |  |
| N stage, n (%) | N0 | 333 (33.4%) | 316 (31.7%) | 0.003 |
|  | N1 | 91 (9.1%) | 135 (13.6%) |  |
|  | N2 | 68 (6.8%) | 46 (4.6%) |  |
|  | N3 | 3 (0.3%) | 4 (0.4%) |  |
| M stage, n (%) | M0 | 359 (45.6%) | 396 (50.3%) | 0.433 |
|  | M1 | 18 (2.3%) | 14 (1.8%) |  |
| Pathologic stage, n (%) | Stage I | 272 (27.1%) | 246 (24.6%) | 0.113 |
|  | Stage II | 125 (12.5%) | 158 (15.8%) |  |
|  | Stage III | 82 (8.2%) | 86 (8.6%) |  |
|  | Stage IV | 19 (1.9%) | 14 (1.4%) |  |
| Gender, n (%) | Female | 242 (23.9%) | 164 (16.2%) | < 0.001 |
|  | Male | 265 (26.1%) | 343 (33.8%) |  |
| Smoker, n (%) | No | 66 (6.7%) | 26 (2.6%) | < 0.001 |
|  | Yes | 427 (43.2%) | 469 (47.5%) |  |
| Age, meidan (IQR) |  | 67 (60, 73) | 67 (60, 73) | 0.984 |
